# Retinocytoma: understanding pathogenesis, diagnosis, and treatment approaches

**DOI:** 10.1186/s40942-025-00642-z

**Published:** 2025-02-25

**Authors:** Maysa Al-Hussaini, Sarah Al Sharie, Hala Sultan, Mona Mohammad, Yacoub A. Yousef

**Affiliations:** 1 Department of Cell Therapy and Applied Genomics, King Hussein Cancer Center, 11941 Amman, Jordan; 2 Department of Pathology and Laboratory Medicine, King Hussein Cancer Center, 11941 Amman, Jordan; 3 Office of Scientific Affairs and Research, King Hussein Cancer Center, 11941 Amman, Jordan; 4 Ophthalmology, Department of Surgery, King Hussein Cancer Center, 11941 Amman, Jordan

**Keywords:** Retinocytoma, Retinoma, Photoreceptor cells, *RB1* gene, Immunohistochemistry, Ocular tumors.

## Abstract

Retinocytoma, or retinoma, is a rare benign intraocular tumor primarily affecting the retina. It is often considered a precursor or a differentiated form of retinoblastoma, a malignant retinal tumor predominantly seen in children. Despite its non-aggressive nature and excellent prognosis, retinocytoma remains a significant area of interest due to its implications for genetic counseling, early detection, and management of ocular tumors. The pathogenesis of retinocytoma is closely linked to mutations in the *RB1* gene, which plays a vital role in regulating the cell cycle. The detection of *RB1* mutations in peripheral blood indicates germline disease, substantially elevating the risk of bilateral retinoblastoma development. Despite its benign nature, retinocytoma necessitates vigilant monitoring due to its potential to transform into retinoblastoma. Current treatment strategies primarily focus on observation and regular follow-up. However, more aggressive treatments are considered if malignant transformation is suspected. The prognosis is generally favorable, with most patients maintaining good visual acuity and a low risk of progression to retinoblastoma. We aim to present an up-to-date review on epidemiology, clinical features, pathogenesis, macroscopic and histopathological features, diagnostic criteria, prognosis, and management strategies.

## Introduction

Retinocytoma, also known as retinoma, is a rare and benign intraocular tumor that primarily affects the retina [1]. It is often considered a precursor or a differentiated form of retinoblastoma, a malignant retinal tumor most commonly seen in children [2]. The term retinoma was first coined by Gallie et al. to replace the older term “spontaneously regressing retinoblastoma.” [1]. The term retinocytoma was later proposed following the terminology for pineal gland tumors [3, 4]. The earliest description of “spontaneously regressing retinoblastoma” was published in 1956 [5]. At that time, compiled evidence of regression included four points: [1] a family history of the disease (which might not be present in all cases) [2], clinical diagnosis of bilateral retinoblastoma with removal of one eye (usually with histological proof) followed by arrest of growth in the other eye [3], a fundus image corresponding to that of verified cases, and [4] calcified tumor cells in phthisical eyes [5].

According to the International Classification of Diseases for Oncology (ICD-O), retinocytoma is coded as 9510/0 (Retinocytoma, C69.2), while retinoblastoma that has spontaneously regressed is coded as 9514/1 (Retinoblastoma, spontaneously regressed, C69.2) [6]. In the International Classification of Diseases, 11th Revision (ICD-11), retinocytoma is coded as XH5AV1 (Retinocytoma, neuroepitheliomatous neoplasm) and is also classified under 2F36 (Benign neoplasm of eye or ocular adnexa) [7].

## Methodology

This narrative review was conducted using the SANRA (Scale for Assessing Narrative Review Articles) criteria to ensure a structured approach in collecting, evaluating, and synthesizing the existing literature on retinocytoma [8].

A comprehensive search of electronic databases, including PubMed, MEDLINE, and Embase, was performed to identify relevant research published up to January 2023. The search strategy utilized a combination of keywords such as “retinocytoma,” “retinoma,” “RB1 gene mutation,” “ocular tumors,” and “retinal neoplasms.” To ensure comprehensiveness, reference lists of key articles were also reviewed to capture any additional relevant studies. The inclusion criteria were carefully defined to focus on studies providing substantial insights into retinocytoma. Articles included peer-reviewed publications and case studies detailing the clinical features, diagnostic methodologies, pathogenesis, prognosis, or management strategies of retinocytoma. In addition to research discussing imaging techniques, histopathological findings, or genetic analysis relevant to retinocytoma. Articles that exclusively focused on retinoblastoma without discussing retinocytoma, or published in languages other than English, were excluded.

The findings were synthesized using a thematic analysis framework. Publications were grouped into common themes, facilitating the development of a coherent narrative. This approach allowed the review to trace advancements in understanding retinocytoma, including its genetic basis, clinical presentation, and management. The narrative further highlighted the significance of innovations in imaging techniques and genetic testing in diagnosing and monitoring this rare condition.

A narrative review was chosen over a systematic review due to the sparse and heterogeneous nature of the available literature on retinocytoma. This approach enabled a broad exploration of current knowledge, identification of research gaps, and the presentation of a synthesized account that provides a foundation for future studies.

## Epidemiology

Retinocytoma is considerably less common than retinoblastoma. The exact prevalence is difficult to ascertain due to its rarity and often asymptomatic nature. Many cases are incidentally discovered during routine eye examinations or screenings in families with a history of retinoblastoma [9]. However, according to available reports, retinocytoma accounts for 1.8-3.0% in USA [10, 11], 3.2% in Greece [12], and up to 11% in Switzerland of retinoblastoma patients and their families [13]. This wide variability in reported incidence between studies (1.8–11%) could be explained by geographic variation and diagnostic differences. A study by Singh et al. suggested that differences in access to healthcare and genetic predisposition among populations may explain the variation [10]. Variation in defining and diagnosing retinocytoma may also play a role [11]. Furthermore, advancements in imaging technologies, such as optical coherence tomography (OCT) and autofluorescence, have enhanced the identification of subtle lesions that might previously have gone unnoticed, thereby increasing the reported incidence. In histologically examined enucleated eyes, however, the reported incidence varies between 15.6 and 20.4% [14]. There is a slight predominance in males (53-58%), with a clear predilection for whites (82-85%) [10, 11]. Moreover, a family history of retinoblastoma is reported in approximately 13% of retinocytoma cases [15].

## Clinical features including examination

Retinocytoma is typically identified in patients with a known family history of retinoblastoma or through screening of individuals at risk for retinoblastoma [16]. Most patients (82%) are older than five years at the time of presentation [10], and the median age at diagnosis varies between 5 years to 15 years (range, 4–45 years) [10, 11]. The average age range is 23 [13] to 28.7 years [12]. The oldest patient described is an 88-year-old [11]. Many patients (41%) do not have symptoms, and visual acuity is normal in the majority [10]. Most cases are discovered incidentally in parents and relatives of children with retinoblastoma [17, 18]. Retinocytoma can occur in association with retinoblastoma in the same or the fellow eye [13]. It can also be discovered when an overlying retinoblastoma is treated or in association with phthisis bulbi, which can only be linked to retinoblastoma if other evidence, including genetic testing, is present [13, 19, 20]. When symptomatic, common symptoms include blurred vision (24%) and strabismus (18%) [10]. Cases presenting with exotropia, esotropia, and floaters secondary to vitreous seeding have been rarely reported [21–24]. A single case of swelling cataract and phacomorphic glaucoma in a patient with retinocytoma is described [25]. Leukocoria, a frequent presentation in retinoblastoma, is seen in younger patients [11]. Retinocytoma is unresponsive to chemotherapy, a feature that should raise suspicion of the diagnosis [26, 27]. It also does not show evidence of growth over time [26, 27].

Retinocytoma is most commonly unilateral, but 13% of cases can be bilateral [10, 13]. It is rare to have trilateral disease or a second malignancy [13, 28, 29]. The tumors are usually small in size, with a median basal dimension of 6.0–6.25 mm (range, 0.3–15.0 mm) and a median thickness of 1.75–2.3 mm (range, 0.05–5.0 mm) [10, 11].

**Fig. 1:**
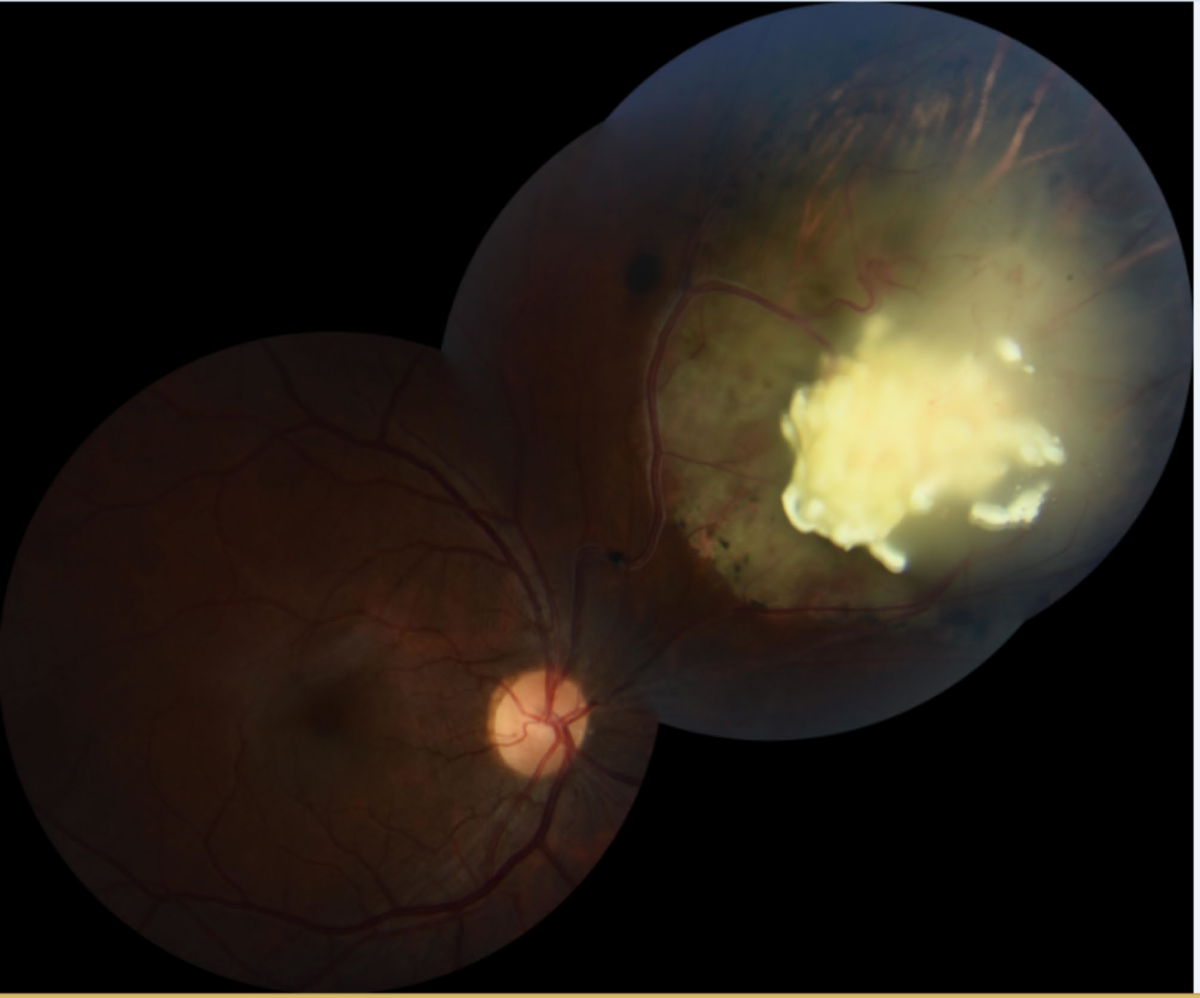
Color fundus photograph of the right eye in a 30-year-old female, who was incidentally found to have a superonasal retinocytoma. The calcified tumor is endophytic, with surrounding patch of chorio-retinal atrophy

Under indirect ophthalmoscopy, retinocytoma exhibits features resembling those of a partially treated retinoblastoma in an untreated eye. There are four clinical features of retinocytoma. These include the presence of a translucent retinal mass (88% of cases), intralesional calcification (63%), retinal pigment epithelial changes/disturbance/clumping (54%), and chorioretinal atrophy (54%) with or without staphyloma [10]. Any combination of two or three of the aforementioned is present in 83% and 46% of cases, respectively [10]. Occasional cases with calcified vitreous deposits/seeding are also reported [23, 30–32]. An intratumoral cystic pattern is rarely described (5.8%) [10]. Most cases are extra-macular [10]. Exophytic and endophytic pre-regression growth patterns have also been described [12]. Figure 1 demonstrates an example of a color fundus photograph for an incidentally diagnosed retinocytoma.

**Fig. 2:**
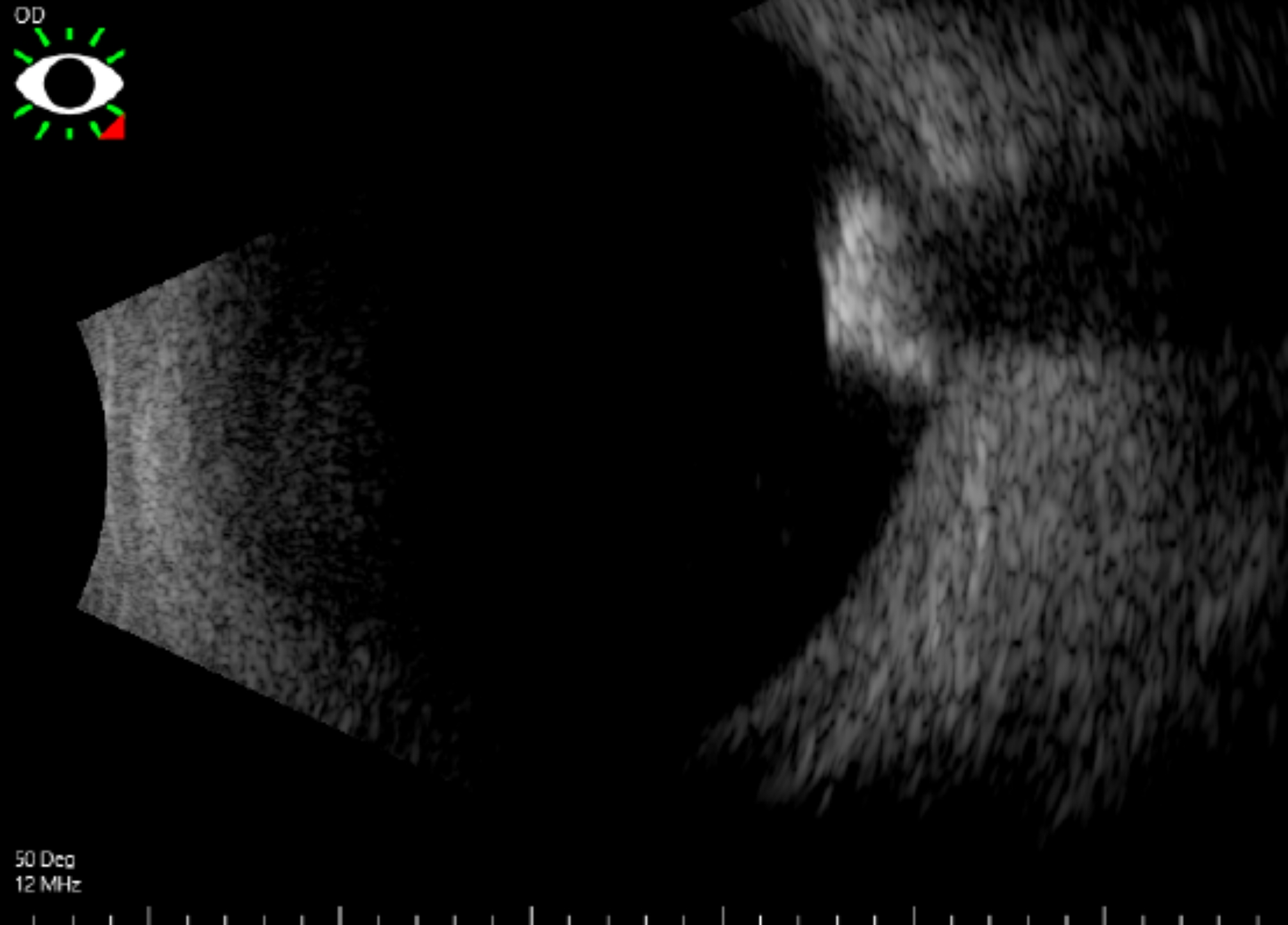
B-scan ultrasonography of the right eye showing a retinocytoma mass with calcifications, exhibiting high internal reflectivity and associated shadowing

Ultrasonography facilitates tumor size assessment and delineates calcified lesions, characterized by acoustic solidity and shadowing resulting from intralesional calcification. A-scan ultrasonography shows a sharp anterior border, high internal reflectivity, and attenuation of orbital echoes posterior to the tumor [23, 33]. Figure 2 demonstrates an example of B-scan ultrasonography of the right eye with retinocytoma.

In optical coherence tomography (OCT), a hyperreflective layer analogous to the preserved nerve fiber layer overlying the lesion, as well as symmetrical hypo-reflective spaces in the underlying choroid with sudden disruption of the choroid in areas of choroidal atrophy, are described [34]. Vitreous seeding in retinocytoma can also be detected using OCT [31]. However, OCT alone cannot differentiate between retinoblastoma and retinocytoma [35].

At early stages, retinoblastoma presents as small, intra retinal tumors with early calcifications and feeder vessels, while retinocytoma appears as a stable, translucent mass with limited vascularization [36]. At advanced stages, retinoblastoma demonstrates rapid growth, extensive calcifications, subretinal fluid, associated with sub-retinal and vitreous seeding, whereas retinocytoma remains non-progressive, with well-demarcated calcifications and no invasive features [37].

In recent years, swept-source OCT (SS-OCT) and OCT angiography (OCTA) have emerged as valuable tools in supporting the diagnosis of retinocytoma [22]. SS-OCT in retinocytoma often reveals significant thinning of the posterior ocular coat due to atrophic retinochoroidal layers, while OCTA, leveraging its three-dimensional imaging capabilities, typically demonstrates a well-defined superficial retinal plexus but may encounter limitations in assessing the deeper retinal and choroidal plexuses due to distortion and projection artifacts caused by calcifications and scar tissue [22]. Fundus autofluorescence can show features similar to pre-treated RB [34, 38]. In retinocytoma, it shows a mixed pattern of hypoautofluorescence, isoautofluorescence, and hyperautofluorescence, with hypoautofluorescence in peripheral areas due to retinal pigment epithelium atrophy, isoautofluorescence and hyperautofluorescence in the central lesion, and hyperautofluorescence near areas of chorioretinal atrophy or exposed sclera from unmasking of scleral autofluorescence, resembling features seen in pre-treated retinoblastoma [34, 38].It also shows features of tumor regression or arrest, including calcification, retinal pigment epithelium defects, lesion translucence, and chorioretinal atrophy [34, 38]. OCTA and multicolor imaging scans are also crucial in establishing the diagnosis and in identifying malignant transformation [39]. Multicolor imaging in retinocytoma shows central areas of hyperreflectivity caused by calcification, with bulging and calcification at different levels in the retina [39]. It also highlights chorioretinal atrophy and exposed sclera, especially on infrared reflectance channels. OCTA shows distorted retinal vessels over calcified areas but has difficulty assessing deeper plexuses due to projection artifacts. It can also detect malignant changes by identifying new blood vessels and connections between retinal and tumor vessels. The choroidal vessels are visible in areas with retinal pigment epithelial atrophy [39]. Fluorescein angiography reveals tortuous, sclerosed feeder vessels and highlights specific features of retinocytoma, including limited intrinsic vascularity, a feeder artery of normal caliber, surrounding window defects, and minimal or absent dye leakage into the vitreous cavity [40]. In comparison, fluorescein angiography in retinoblastoma typically shows rapid filling of a feeder artery, a complex intratumoral vascular network, progressive hyperfluorescence due to intense tumor staining, and dye leakage into the vitreous cavity [41].

Magnetic resonance imaging (MRI) can also be helpful depending on T1 and T2 image characteristics, but generally, retinocytoma may be indistinguishable from retinoblastoma which appears as solid intraocular masses with intermediate to high signal intensity on T1-weighted images and low to intermediate signal intensity on T2-weighted images [42, 43].

The primary differential diagnosis for retinocytoma is retinoblastoma, mainly since both conditions can occur in the same patient or family [44]. Features that help distinguish retinocytoma from retinoblastoma include the minimal vascular proliferation, and the absence of symptoms [3]. Retinoblastoma generally does not show chorioretinal atrophy but does present with retinal pigment epithelium (RPE) alterations. Furthermore, prominent feeder vessels are commonly seen in retinoblastoma, although exudation is notably absent [45].

Other important considerations in the differential diagnosis include astrocytic hamartoma, which is commonly associated with tuberous sclerosis and typically presents as yellow, spherical lesions that may exhibit calcifications. Unlike retinocytoma, astrocytic hamartomas lack chorioretinal atrophy and retinal pigment epithelial (RPE) changes. However, not all astrocytic hamartomas are calcified, and while they can grow, their progression is typically slow and non-aggressive, distinguishing them from malignant retinal tumors such as retinoblastoma. Additionally, astrocytic hamartomas do not have prominent feeder vessels, though exudation may occasionally be present. Importantly, unlike astrocytic hamartomas, retinocytomas has no recognized associations with tuberous sclerosis or neurofibromatosis [46].

Other conditions that may mimic retinocytoma include congenital hypertrophy of the retinal pigment epithelium (CHRPE), and retinal hemangioblastoma [45, 47–49]. Both retinocytoma and CHRPE can present as well-defined, flat, pigmented lesions on fundus examination [50]. Retinocytoma may show areas of calcification and chorioretinal atrophy, which can sometimes mimic the uniform pigmentation and surrounding halo effect seen in CHRPE [50]. However, CHRPE is typically non-calcified, stable over time, and lacks intrinsic vascularity or growth potential, helping to distinguish it from retinocytoma, which may display subtle vascular changes or calcifications on imaging [50].

## Pathogenesis and etiology

The pathogenesis of retinocytoma is predominantly linked to mutations in the *RB1* gene, a tumor suppressor gene located on chromosome 13q14 [51]. The *RB1* gene plays a critical role in regulating the cell cycle by encoding the retinoblastoma protein (pRB), which controls the progression from the G1 to the S phase [52]. In its active form, pRB binds to and inhibits E2F transcription factors, thereby preventing uncontrolled cell proliferation [52].

**Fig. 3:**
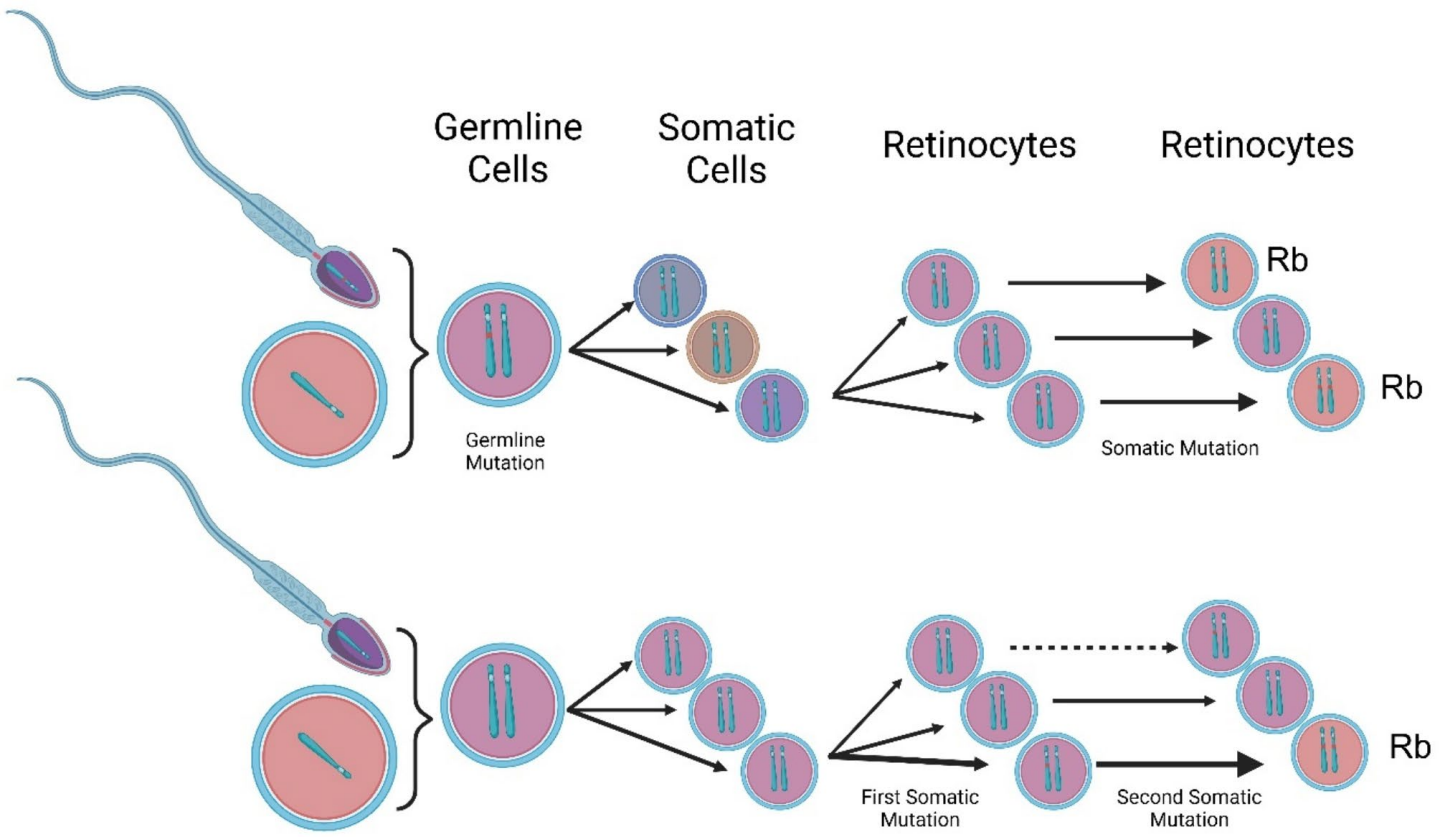
Knudson’s two-hit hypothesis. If the first mutation is present in the germline, all somatic cells will carry one mutant allele. This increases the probability of a second mutation occurring during the somatic stage, which can affect multiple retinocytes in both eyes and other somatic precursor cells. If both mutations occur during the somatic stage, it leads to the development of unilateral unifocal retinoblastoma. Figure generated using BioRender

Retinoblastoma and retinocytoma share the same genetic changes, although retinocytoma shows low penetrance to the *RB* gene [53]. Knudson’s two-hit hypothesis states that both alleles of the *RB1* gene must be mutated to convert normal retinal cells into neoplastic retinoblastoma cells. Figure 3 illustrates the mechanism of *RB1* mutation based on Knudson’s two-hit hypothesis. The presence of *RB1* mutations impairs the normal regulatory function of pRB, resulting in deregulated cell cycle progression and potential tumor formation [54]. However, unlike retinoblastoma, retinocytoma might stop at the M2 level and might not transform into retinoblastoma [55]. It could also arise if the second hit occurs at a later stage of cell maturation, when the precursor cell has limited mitotic capability and is unable to sequentially accumulate additional mutations [56, 57].

The inactivation of the *RB1* gene leads to disruption of several vital cellular pathways; Firstly, the loss of functional pRB allows E2F transcription factors to remain active, promoting the expression of genes required for DNA synthesis and cell cycle progression. This uncontrolled cell division is a hallmark of tumorigenesis [58]. It is possible that proteins encoded by *RB1* gene variants retain part of the wild-type protein function. Therefore, in the presence of a partially functional RB1 protein, the precursor cells form a retinocytoma instead of a retinoblastoma. Also, the *RB1* gene plays a role in apoptosis, the programmed cell death mechanism eliminates damaged or abnormal cells [59]. In retinocytoma, impaired pRB function can disrupt apoptotic pathways, allowing the survival and accumulation of aberrant cells [60]. This is reflected in the tumor’s histopathological appearance, where the retinal layers are relatively preserved, and the cells exhibit less aggressive behavior [61]. Recent evidence suggests that genetic instability and aneuploidy are key factors distinguishing retinoblastoma from retinocytoma suggesting that retinocytoma is genetically a precursor of retinoblastoma [62–64]. A study by Dimaras et al. observed that retinomas, with their high expression of senescence-associated proteins like p16INK4a and p130, exhibit low-level genomic instability [62]. They observed that this instability, which is initially kept in check by cellular senescence, can escalate, leading to the malignant transformation to retinoblastoma. Moreover, based on molecular analysis, they suggested a clonal relationship between retinocytoma, and retinoblastoma based on the *RB1* gene mutation, underscoring the role of retinoma as a precursor lesion that, upon overcoming the senescence barrier, can evolve into highly proliferative and malignant retinoblastoma.

Also, epigenetic modification, through *RB1* promoter hypermethylation, might result in phenotypically variable tumor expression resulting in retinocytoma [65]. Despite the common genetic links between retinocytoma and retinoblastoma, the reasons why some individuals develop retinocytoma rather than retinoblastoma remain unclear [66]. Dryja et al. proposed another hypothesis suggesting that retinocytoma may result from low penetrance of retinoblastoma [67].

## Macroscopic and histopathological features

Macroscopically, retinocytoma appears as a translucent, slightly elevated retinal mass with well-defined borders [68]. The lesion often exhibits a lobulated or map-like pattern, contributing to its similar appearance to retinoblastoma [69]. One of the hallmark macroscopic features is the presence of calcific foci, which appear as chalky-white patches within the tumor [37].

**Fig. 4:**
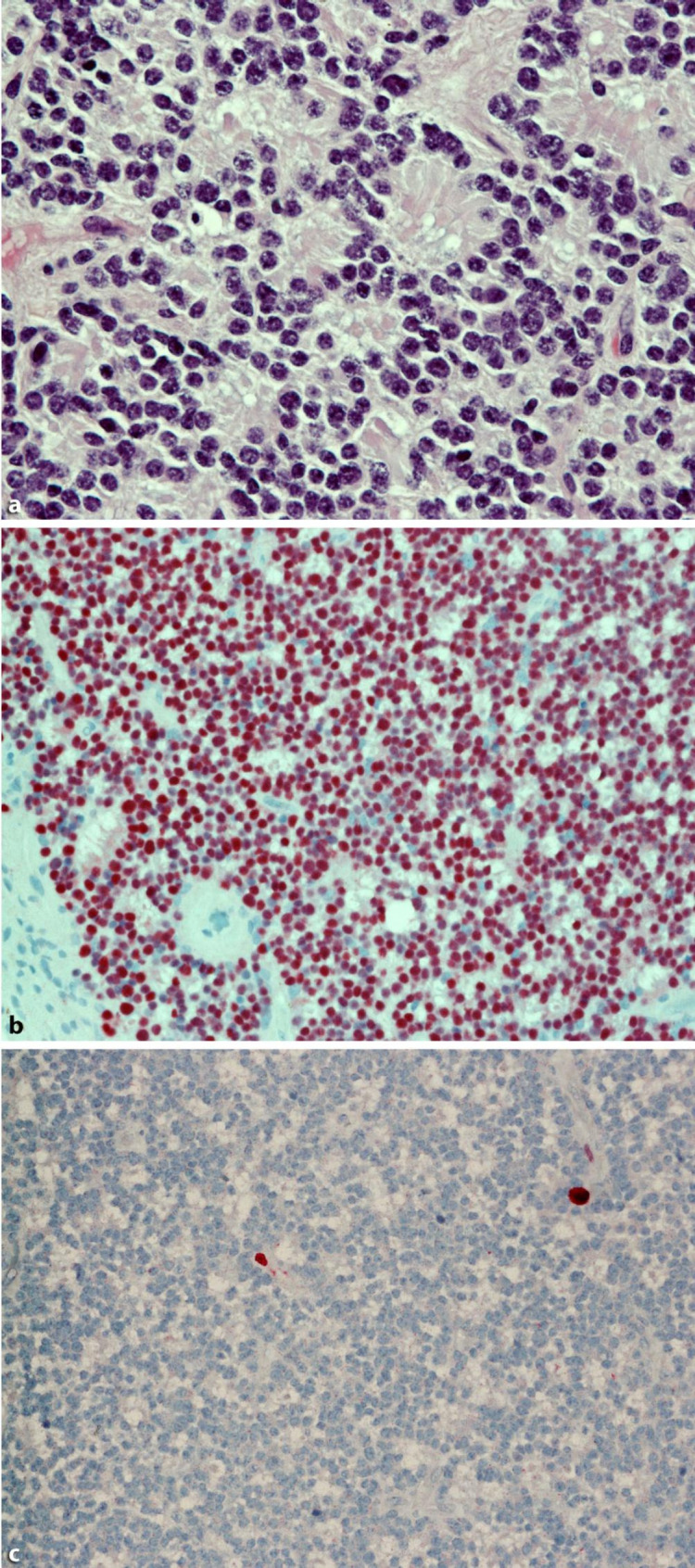
Retinocytoma (Retinoma): Photoreceptor differentiation with numerous fleurettes (**a**, HE stain, original magnification 40x). Nuclear positivity for CRX (**b**, CRX, original magnification 20x). Negativity for Ki67 with a few reactive control cells (**c**, Ki67, original magnification 20x). Figure obtained with permission from Metz et al. (2017) [93]

Characteristically, retinocytoma displays benign histopathologic features. This includes bland round to oval nuclei with evenly dispersed chromatin, eosinophilic, occasionally clear cytoplasm with distinct cell borders, scattered fleurettes as evidence of prominent photoreceptor differentiation, a fibrillar eosinophilic stroma composed of elongated cytoplasmic processes, calcific foci within viable tumor, with no evidence of rosettes, mitoses, nuclear atypia, pleomorphism, or necrosis [62, 70, 71]. There is a well-vascularized ground substance with calcific foci, as well as RPE hyperplasia [71]. Multinucleate tumor giant cells have been occasionally described [72]. Figure 4 demonstrates the histopathological features of retinocytomas.

Usually, there is no evidence of retinal detachment [4]. The architecture of the normal retina is, however, obliterated. There is no evidence of invasion of the choroid, the optic nerve, or the vitreous [73].

Furthermore, evidence of retinocytoma can be seen in eyes enucleated for retinoblastoma [74]. Around 20% of retinoblastoma cases show evidence of photoreceptor differentiation, commonly localized to the base of the tumor, supporting the notion that retinocytoma might be a precursor lesion to retinoblastoma [74].

Immunohistochemically, retinocytoma cells are positive for anti-RB antibodies, retinal S antigen, S-100 protein, and glial fibrillary acidic protein (GFAP) [70, 75, 76]. P16 (INK4a) immunostains are negative in areas of retinocytoma exhibiting fleurettes differentiation [77]. By electron microscopy, photoreceptor differentiation in the cytoplasm of tumor cells is depicted [4].

Retinoblastoma is the most important pathological differential diagnosis. In contrast to retinocytoma, it shows the proliferation of malignant cells with pleomorphism, mitosis, and necrosis [62, 70, 71]. Homer-Wright and Flexner-Wintersteiner rosettes are frequently seen [73]. P16 immunostain tends to be positive in retinoblastoma, especially in poorly differentiated tumors [77].

## Prognosis and prediction of retinocytoma

Retinocytoma can remain stable with no evidence of progression over an extended period [78]. However, risk of malignant transformation to retinoblastoma is reported, and ranges between 4% [10] to 12% [12], a tendency which appears to increase over time and reaches up to 15.3% by 10–20 years [11]. It appears that the only factor predictive of transformation is increasing thickness [36]. This tendency might be a plausible explanation for cases of retinoblastoma developing in adults [79].

In a comprehensive study spanning 20 years, Shields et al. explored the clinical features and long-term outcomes of retinocytoma in 62 patients [11]. The study included 78 tumors and reported that 3% of the 2,021 retinoblastoma patients had retinocytoma, with a median age of 5 years at presentation. Notably, malignant transformation of retinocytoma into retinoblastoma was documented in 2.7% of cases by two years, 9.2% by five years, and 15.3% by 10–20 years. Multivariate analysis identified increasing tumor thickness as the only predictor of transformation into retinoblastoma. Tumors that transformed had a mean thickness of 4.3 mm, compared to 2.4 mm in those that did not (*p* = 0.003). Each 1 mm increase in thickness corresponded to a hazard ratio of 2.83 for transformation. The average time to transformation was 9.8 years (117 months), with a range from 1.6 to 23.6 years.

Therefore, patients with the diagnosis of retinocytoma should be offered a lifelong close observation, and genetic counseling [80]. In addition, genetic counseling and frequent observation should be offered to their offspring. Genetic testing from blood [59] or saliva sample [33] can be used for the detection of germline *RB1* gene mutation to establish the diagnosis, which appears to be more common in young patients [11]. Two patterns of reactivation have been described in the literature: benign cystic growth or malignant transformation [12], which can be rapid with vitreous seeding [10, 70]. Furthermore, ophthalmic examinations of parents and siblings of individuals with retinoblastoma should always be conducted to rule out the presence of retinoblastoma or asymptomatic retinocytoma [17, 81]. Occasional cases of retinocytoma might regress spontaneously [82]. However, aggressive endo- and exophytic growth prior to spontaneous regression have been reported [12].

## Management of retinocytoma

The management of retinocytoma focuses on regular monitoring, early detection of potential malignant transformation, and intervention when necessary to preserve vision and prevent complications [83]. Due to its benign nature, the primary approach involves vigilant observation and genetic counseling [11, 83].

Initial management begins with a comprehensive ophthalmic examination, including fundoscopy to identify the characteristic features of retinocytoma, such as a translucent retinal mass, calcific foci, and RPE changes [68, 84]. Detailed medical and family histories are also essential. Imaging studies, including OCT, B-scan ultrasonography, and brain and orbit MRI, help assess the tumor’s size, location, and structural characteristics, documenting any signs of chorioretinal atrophy or calcification [23, 33, 42, 85, 86]. Genetic testing for *RB1* gene mutations using blood or saliva samples is conducted, employing methods such as Sanger sequencing to detect specific mutations [33, 59, 87].

Genetic counseling for patients and their families is an integral part of management. It explains the nature of retinocytoma, the risk of malignant transformation, and the importance of regular monitoring [33, 59, 87]. Counseling also covers the hereditary aspects of *RB1* mutations and their implications for family members. Surveillance of family members, including ophthalmic examinations of parents and siblings, is necessary to detect any signs of retinocytoma or retinoblastoma. Genetic testing for family members can identify carriers of *RB1* mutations [33, 59, 87].

For stable retinocytomas with no signs of progression, continued observation with regular follow-up is the primary management strategy [10, 11]. One study suggests an observation protocol that involves close monitoring initially on a monthly basis, transitioning to every two months once stability is confirmed. Over time, the interval is gradually extended to every four months, and subsequently every six months. Long-term, continued observation every six months lifelong to ensure ongoing tumor stability is recommended [11]. Advanced imaging techniques, such as OCTA and SS-OCT, are invaluable in monitoring retinocytoma progression. These modalities provide detailed visualization of tumor vascularity, thickness, and adjacent retinal structures, which can aid in identifying subtle changes suggestive of transformation [22].

Laser therapy or cryotherapy may be considered in specific cases, such as for tumors impacting vision, those demonstrating early signs of malignant transformation, or if complications such as vitreous hemorrhage occur, with the choice of therapy tailored to the individual clinical scenario and patient-specific factors [36, 88, 89]. In cases of confirmed malignant transformation to retinoblastoma, the treatment should be customized based on site of the tumor, extension, and thickness [36]. Treatment options include combination of systemic chemotherapy with focal consolidation therapy, intra-arterial chemotherapy, (which is an effective treatment option, delivering chemotherapy directly to the tumor and minimizing systemic side effects), Transpupillary thermotherapy laser, and radioactive plaque brachytherapy [90, 91].

A study by Kiratli et al. [92] reported two cases of retinocytoma, which were initially managed by observation to monitor tumor progression. In both cases, intra-arterial chemotherapy with 7.5 mg melphalan was administered, resulting in the rapid regression of the new developed growths while the original tumors remained unchanged. This approach successfully stabilized both patients’ condition and visual acuity over the follow-up periods. Moreover, Yaman et al. reported a case involving a 12-year-old girl who underwent a single session of transpupillary thermotherapy laser to prevent malignant transformation [16]. During a 3.5-year follow-up period, the lesion’s dimensions and the patient’s visual acuity remained stable without additional treatment.

Occasional cases of retinocytoma may also result in vitreous hemorrhage necessitating vitrectomy [89], as long as there is no active intraocular retinoblastoma. In a case reported by Grassi et al. [89], a 35-year-old man with bilateral retinocytoma experienced persistent vitreous hemorrhage in his left eye for six months caused by an incomplete posterior vitreous detachment and was managed by a 23G pars plana vitrectomy, combined with endolaser and endodiathermy to treat the bleeding retinal vessel. This surgical procedure successfully cleared the hemorrhage and restored his visual acuity to 20/15 within six months.

## Conclusion

Retinocytoma, a benign retinal tumor, is typically associated with a favorable prognosis; however, its potential to transform into retinoblastoma underscores the need for lifelong surveillance. Early detection and routine follow-up are critical to preserving optimal visual outcomes and promptly identifying signs of malignant transformation, which, although uncommon, may become more likely over time.

Genetic counseling and testing play a role in managing retinocytoma, especially for individuals with a family history of retinoblastoma. Advances in diagnostic imaging and genetic testing have improved the ability to detect and monitor the tumor effectively.

Management primarily involves vigilant observation, with intervention strategies such as laser therapy, cryotherapy, systemic chemotherapy, and intra-ophthalmic arterial chemotherapy reserved for cases showing signs of malignant transformation. This comprehensive approach ensures early intervention, maintaining vision, and mitigating the risks of progression to retinoblastoma.

## Data Availability

No datasets were generated or analysed during the current study.

## Abbreviations

RB1: Retinoblastoma 1 (gene)
pRB: Retinoblastoma protein
OCT: Optical Coherence Tomography
SS-OCT: Swept-Source Optical Coherence Tomography
OCTA: Optical Coherence Tomography Angiography
MRI: Magnetic Resonance Imaging
ICD-O: International Classification of Diseases for Oncology
ICD-11: International Classification of Diseases 11th Revision
CHRPE: Congenital Hypertrophy of the Retinal Pigment Epithelium
RPE: Retinal Pigment Epithelium
GFAP: Glial Fibrillary Acidic Protein
HE: Hematoxylin and Eosin (stain)
CRX: Cone-Rod Homeobox (gene)
Ki67: A cellular marker for proliferation

## References

[1] Gallie BL, Ellsworth RM, Abramson DH, Phillips RA. Retinoma: spontaneous regression of retinoblastoma or benign manifestation of the mutation? Br J Cancer. 1982;45(4):513–21. 7073943 10.1038/bjc.1982.87 PMC2010981

[2] Archer TC, Sengupta S, Pomeroy SL. Chapter 50 - brain cancer genomics and epigenomics. In: Geschwind DH, Paulson HL, Klein C, editors. Handbook of clinical neurology. Volume 148. Elsevier; 2018. pp. 785–97. 10.1016/B978-0-444-64076-5.00050-8 29478614

[3] Singh AD, Balmer A, Munier F. CHAPTER 80 - retinocytoma or retinoma. In: Singh AD, Damato BE, Pe’er J, Murphree AL, Perry J, editors. Clinical Ophthalmic Oncology. Edinburgh: W.B. Saunders; 2007. pp. 487–90.

[4] Margo C, Hidayat A, Kopelman J, Zimmerman LE. Retinocytoma: a Benign variant of Retinoblastoma. Arch Ophthalmol. 1983;101(10):1519–31. 6626001 10.1001/archopht.1983.01040020521003

[5] Arnold EL, Smith JL, Steward JK. Spontaneous regression of retinoblastoma. Br J Ophthalmol. 1956;40(8):449–61. 13364166 10.1136/bjo.40.8.449 PMC1324645

[6] WHO. International Classification of Diseases for Oncology. 2013 [Available from: https://iris.who.int/bitstream/handle/10665/96612/9789241548496_eng.pdf

[7] WHO. International Classification of Diseases 11th Revision 2019 [Available from: https://icd.who.int/en

[8] Baethge C, Goldbeck-Wood S, Mertens S. SANRA-a scale for the quality assessment of narrative review articles. Res Integr Peer Rev. 2019;4:5. 30962953 10.1186/s41073-019-0064-8 PMC6434870

[9] Bowen RC, Stathopoulos C, Munier FL, Singh AD. Retinocytoma or Retinoma. In: Berry JL, Kim JW, Damato BE, Singh AD, editors. Clinical Ophthalmic Oncology: Retinoblastoma. Cham: Springer International Publishing; 2019. pp. 99–105.

[10] Singh AD, Santos MCM, Shields CL, Shields JA, Eagle RC. Jr. Observations on 17 patients with Retinocytoma. Arch Ophthalmol. 2000;118(2):199–205. 10676785 10.1001/archopht.118.2.199

[11] Shields CL, Srinivasan A, Lucio-Alvarez JA, Shields JA. Retinocytoma/retinoma: comparative analysis of clinical features in 78 tumors and rate of transformation into retinoblastoma over 20 years. J Aapos. 2021;25(3):147. e1-.e8. 10.1016/j.jaapos.2020.11.024 34051357

[12] Abouzeid H, Balmer A, Moulin AP, Mataftsi A, Zografos L, Munier FL. Phenotypic variability of retinocytomas: preregression and postregression growth patterns. Br J Ophthalmol. 2012;96(6):884–9. 22328814 10.1136/bjophthalmol-2011-300896

[13] Balmer A, Munier F, Gailloud C. Retinoma. Case studies. Ophthalmic Paediatrics Genet. 1991;12(3):131–7. 10.3109/13816819109029394 1754160

[14] Dimaras H, Eye. Retinoma Atlas Genet Cytogenet Oncol Haematol.2012-03-01 [Available from: https://atlasgeneticsoncology.org/solid-tumor/5049/eye-retinoma

[15] Singh AD, Santos CM, Shields CL, Shields JA, Eagle RC. Jr. Observations on 17 patients with retinocytoma. Arch Ophthalmol. 2000;118(2):199–205. 10676785 10.1001/archopht.118.2.199

[16] Yaman A, Gündüz K, Saatci O, Koçak N. A rare case of retinocytoma occurring in a 12-year-old child. J Pediatr Ophthalmol Strabismus. 2008;45(1):49–50. 18286965 10.3928/01913913-20080101-21

[17] Saiju R, Duwal S. Bilateral retinoblastoma in early infancy. Nepal J Ophthalmol. 2013;5(1):124–8. 23584660 10.3126/nepjoph.v5i1.7840

[18] Onadim Z, Hykin P, Hungerford J, Cowell JKJB. Genetic counselling in retinoblastoma: importance of ocular fundus examination of first degree relatives and linkage analysis. Br J Ophthalmol. 1991;75(3):147–50. 2012779 10.1136/bjo.75.3.147 PMC1042293

[19] Zhang Q, Chen Y, Wu Z, Ma Q, Zeng R, Guo X, et al. Retinoma and phthisis bulbi of retinoblastoma. 1. Clinical and genetic analysis. Yan Ke Xue bao = Eye Sci. 1992;8(3):117–21. 1303868

[20] Gallie BL, Phillips RA, Ellsworth RM, Abramson DHJO. Significance of retinoma and phthisis bulbi for retinoblastoma. Ophthalmology. 1982;89(12):1393–9. 7162783 10.1016/s0161-6420(82)34622-9

[21] Naseripour M, Akbarzadeh S. Retinocytoma Associated with bilateral Retinoblastoma %J Bina Journal of Ophthalmology. Indian J Ophthalmol. 2007;13(1):130–2. 10.4103/0301-4738.60094 PMC2854451 20195043

[22] Pujari A, Azad SV, Meel R, Lomi NJBCRC. Bilateral retinocytoma: multimodal imaging assessment. BMJ case Rep. 2018;11(1):e225908. 30567213 10.1136/bcr-2018-225908 PMC6301439

[23] Garoon RB, Medina CA, Scelfo C, Harbour JW, RETINOCYTOMA WITH VITREOUS SEEDING: NEW INSIGHTS FROM ENHANCED DEPTH IMAGING OPTICAL COHERENCE TOMOGRAPHY AND HIGH-RESOLUTION POSTERIOR SEGMENT ULTRASONOGRAPHY. Retinal Cases Brief Rep. 2021;15(1):68–70. 10.1097/ICB.0000000000000732 29470300

[24] Lueder GT, Héon E, Gallie BL. Retinoma associated with vitreous seeding. Am J Ophthalmol. 1995;119(4):522–3. 7709984 10.1016/s0002-9394(14)71246-2

[25] Krasyuk EY, Ovchinnikov V. Diagnosis of retinocytoma in the eye with swelling mature cataract (clinical case). Saratoc J Med Scient Res. 2020.

[26] Chung J, Turaka K, Shields CL. Retinocytoma shows lack of response to chemoreduction. J Pediatr Ophthalmol Strabismus. 2010;47(Online):e1–3. 21175116 10.3928/01913913-20101217-03

[27] Singh AD, Shields CL, Shields J, Strabismus. Lack of response to chemoreduction in presumed well differentiated retinoblastoma. J Pediatr Ophthalmol Strabismus. 2002;39(2):107–9. 11911540 10.3928/0191-3913-20020301-11

[28] Moll AC, Imhof SM, Bouter LM, Tan KEJO. Second primary tumors in patients with retinoblastoma a review of the literature. Ophthalmic Genet. 1997;18(1):27–34. 9134547 10.3109/13816819709057880

[29] Korswagen LA, Moll AC, Imhof SM, Schouten-van Meeteren AY. A second primary tumor in a patient with retinoma. Ophthalmic Genet. 2004;25(1):45–8. 15255114 10.1076/opge.25.1.45.29006

[30] Hadjistilianou T, De Francesco S, Martone G, Malandrini A. Retinocytoma associated with calcified vitreous deposits. Eur J Ophthalmol. 2006;16(2):349–51. 16703560 10.1177/112067210601600227

[31] Ramasubramanian A, Leverant A. Vitreous seeding in Retinocytoma: importance of Optical Coherence Tomography. J Pediatr Ophthalmol Strabismus. 2020;57(5):340. 32956485 10.3928/01913913-20200722-03

[32] Singh AD, Damato BE, Pe’er J, Murphree AL, Perry J, editors. Clinical Ophthalmic Oncology. Edinburgh: W.B. Saunders; 2007. pp. 599–611.

[33] Paez-Escamilla M, Walter SD, Ramaiya KJ, Harbour JW. Diagnosis of bilateral Retinocytoma in an adolescent patient using Multimodal Imaging and genetic testing. Opthalmic Surg Lasers Imaging Retin. 2018;49(10):812–4. 10.3928/23258160-20181002-11 30395669

[34] Malhotra PP, Bhushan B, Mitra A, Sen A. Spectral-domain Optical Coherence Tomography and Fundus Autofluorescence features in a case of typical Retinocytoma. Eur J Ophthalmol. 2015;25(6):e123–6. 26109020 10.5301/ejo.5000634

[35] Nadiarnykh O, McNeill-Badalova NA, Gaillard M-C, Bosscha M, Fabius AW, Verbraak FD, et al. Optical coherence tomography (OCT) to image active and inactive retinoblastomas as well as retinomas. Acta Ophthalmol. 2019;98:158–65. 31448879 10.1111/aos.14214 PMC7078953

[36] Navaratnam J, Faber R, Eide N, Lund-Iversen M, Garred Ø, Munier FL. Retinocytoma Undergoing Retinoblastoma Transformation in an Adult Patient. Case reports in ophthalmological medicine. 2023;2023:8127245. 10.1155/2023/8127245 PMC10390264 37529687

[37] Shah PK, Narendran V, Manayath GJ, Chowdhary S. Atypical retinocytoma with diffuse vitreous seeds: an insight. Oman J Ophthalmol. 2011;4(2):81–3. 21897624 10.4103/0974-620X.83659 PMC3160075

[38] Dubey D, Shanmugam MP, Singh D. Fundus autofluorescence in retinocytoma. Indian J Ophthalmol. 2022;70(7):2772. 35791248 10.4103/ijo.IJO_3094_21 PMC9426124

[39] Venkatesh R, Agrawal S, Reddy NG, Pereira AJBCRC. Multimodal imaging in a classic case of unilateral retinocytoma. BMJ case Rep. 2021;14(8):e244167. 34353836 10.1136/bcr-2021-244167 PMC8719160

[40] Dubey D, Shanmugam M, Singh D. Fundus fluorescein angiography in a macular retinocytoma. Indian J Ophthalmol. 2022;70(7):2731–2. 35791225 10.4103/ijo.IJO_161_22 PMC9426118

[41] Shields JA, Sanborn GE, Augsburger JJ, Orlock D, Donoso LA. Fluorescein angiography of retinoblastoma. Trans Am Ophthalmol Soc. 1982;80:98–112. 6892130 PMC1312257

[42] de Graaf P, Göricke S, Rodjan F, Galluzzi P, Maeder P, Castelijns JA, et al. Guidelines for imaging retinoblastoma: imaging principles and MRI standardization. Pediatr Radiol. 2012;42(1):2–14. 21850471 10.1007/s00247-011-2201-5 PMC3256324

[43] Benhamou E, Borges J, Tso MO. Magnetic resonance imaging in retinoblastoma and retinocytoma: a case report. J Pediatr Ophthalmol Strabismus. 1989;26(6):276–80. 2621546 10.3928/0191-3913-19891101-06

[44] Singh AD, Retinoblastoma. Evaluation and Differential diagnosis. In: Singh AD, Seregard S, editors. Ocular tumors. Volume 7. S.Karger AG; 2016. p. 0.

[45] Aerts I, Lumbroso-Le Rouic L, Gauthier-Villars M, Brisse H, Doz F, Desjardins L, Retinoblastoma. Orphanet J Rare Dis. 2006;1:31. 16934146 10.1186/1750-1172-1-31 PMC1586012

[46] Martin K, Rossi V, Ferrucci S, Pian D. Retinal astrocytic hamartoma. Optometry. 2010;81(5):221–33. 20435268 10.1016/j.optm.2009.12.009

[47] Reich E, Thaung C, Sagoo MS. Classification of retinal and retinal pigment epithelium tumors. In: Singh AD, Damato BE, editors. Clinical Ophthalmic Oncology: retinal tumors. Cham: Springer International Publishing; 2019. pp. 1–3.

[48] Martin K, Rossi V, Ferrucci S, Pian D. Retinal astrocytic hamartoma. Optometry - J Am Optom Association. 2010;81(5):221–33. 10.1016/j.optm.2009.12.009 20435268

[49] Pineles SL, Balcer LJ. 5 - visual loss: Optic neuropathies. In: Liu GT, Volpe NJ, Galetta SL, editors. Liu, Volpe, and Galetta’s Neuro-Ophthalmology. Third Edition): Elsevier; 2019. pp. 101–96.

[50] Braga CS, Ricardo OMP, Cordeiro FM, Vieira JM, Nogueira FB. Suspect asymptomatic lesions: congenital hypertrophy of the Retinal Pigment Epithelium (CHRPE). Romanian J Ophthalmol. 2021;65(3):275–8. 10.22336/rjo.2021.55 PMC8697784 35036651

[51] Grossniklaus HE, Retinoblastoma. Fifty years of progress. The LXXI Edward Jackson Memorial Lecture. Am J Ophthalmol. 2014;158(5):875–91. 25065496 10.1016/j.ajo.2014.07.025 PMC4250440

[52] Heidemann SR. 2 - Cancer: a Disease of Cellular Proliferation, Life Span, and death. In: Klein BG, editor. Cunningham’s Textbook of Veterinary Physiology (Sixth Edition). St. Louis (MO): W.B. Saunders; 2020. pp. 29–50.

[53] Alekseeva EA, Babenko OV, Kozlova VM, Ushakova TL, Kazubskaya TP, Nemtsova MV et al. Parental origin of the RB1 gene mutations in families with low Penetrance Hereditary Retinoblastoma. Cancers (Basel). 2021;13(20). 10.3390/cancers13205068 PMC8534066 34680218

[54] Stenfelt S, Blixt MKE, All-Ericsson C, Hallböök F, Boije H. Heterogeneity in retinoblastoma: a tale of molecules and models. Clin Translational Med. 2017;6(1):42. 10.1186/s40169-017-0173-2 PMC5680409 29124525

[55] Gallie BL, Campbell C, Devlin H, Duckett A, Squire JA. Developmental basis of retinal-specific induction of Cancer by RB Mutation1. Cancer Res. 1999;59(7Supplement):s1731–5. 10197588

[56] Gallie BL, Dunn JM, Chan HS, Hamel PA, Phillips RA. The genetics of retinoblastoma: relevance to the patient. Pediatr Clin North Am. 1991;38(2):299–315. 2006079 10.1016/s0031-3955(16)38079-8

[57] Mastrangelo D, De Francesco S, Di Leonardo A, Lentini L. Hadjistilianou TJIjoc. Does the evidence matter in medicine? The retinoblastoma paradigm. Int J Cancer. 2007;121(11):2501–5. 17657745 10.1002/ijc.22944

[58] Chen HZ, Tsai SY, Leone G. Emerging roles of E2Fs in cancer: an exit from cell cycle control. Nat Rev Cancer. 2009;9(11):785–97. 19851314 10.1038/nrc2696 PMC3616489

[59] Wu S, Zou X, Sun Z, Zhu T, Wei X, Sui R. Unilateral retinocytoma associated with a variant in the RB1 gene. Mol Genet Genomic Med. 2020;8(4):e1156. 31997559 10.1002/mgg3.1156 PMC7196460

[60] Indovina P, Pentimalli F, Casini N, Vocca I, Giordano A. RB1 dual role in proliferation and apoptosis: cell fate control and implications for cancer therapy. Oncotarget. 2015;6(20). 10.18632/oncotarget.4286 PMC4627222 26160835

[61] Ruijtenberg S, van den Heuvel S. Coordinating cell proliferation and differentiation: antagonism between cell cycle regulators and cell type-specific gene expression. Cell Cycle. 2016;15(2):196–212. 26825227 10.1080/15384101.2015.1120925 PMC4825819

[62] Dimaras H, Khetan V, Halliday W, Orlic M, Prigoda NL, Piovesan B, et al. Loss of RB1 induces non-proliferative retinoma: increasing genomic instability correlates with progression to retinoblastoma. Hum Mol Genet. 2008;17(10):1363–72. 18211953 10.1093/hmg/ddn024

[63] Abouzeid H, Schorderet DF, Balmer A, Munier FL. Germline mutations in retinoma patients: relevance to low-penetrance and low-expressivity molecular basis. Mol Vis. 2009;15:771–7. 19390654 PMC2671583

[64] Mastrangelo D, De Francesco S, Di Leonardo A, Lentini L, Hadjistilianou T. Does the evidence matter in medicine? The retinoblastoma paradigm. Int J Cancer. 2007;121(11):2501–5. 17657745 10.1002/ijc.22944

[65] Mastrangelo D, Loré C, Grasso G. The epigenetic origin of Retinoblastoma. Eur Ophthalmic Rev. 2012;06:130.

[66] Gallie BL, Dunn JM, Chan HSL, Hamel PA, Phillips RA. The Genetics of Retinoblastoma: relevance to the patient. Pediatr Clin North Am. 1991;38(2):299–315. 2006079 10.1016/s0031-3955(16)38079-8

[67] Dryja TP, Rapaport J, McGee TL, Nork TM, Schwartz TL. Molecular etiology of low-penetrance retinoblastoma in two pedigrees. Am J Hum Genet. 1993;52(6):1122–8. 8099255 PMC1682279

[68] Bubshait L, Alburayk K, Alabdulhadi H, Emara K, Retinocytoma. Case Ser Cureus. 2023;15(8):e42958. 37667715 10.7759/cureus.42958 PMC10475323

[69] Balmer A, Munier F. Differential diagnosis of leukocoria and strabismus, first presenting signs of retinoblastoma. Clin Ophthalmol. 2007;1(4):431–9. 19668520 PMC2704541

[70] Eagle RC Jr., Shields JA, Donoso L, Milner RS. Malignant transformation of spontaneously regressed retinoblastoma, retinoma/retinocytoma variant. Ophthalmology. 1989;96(9):1389–95. 2780006 10.1016/s0161-6420(89)32714-x

[71] Aaby AA, Price RL, Zakov ZN. Spontaneously regressing retinoblastomas, retinoma, or retinoblastoma group 0. Am J Ophthalmol. 1983;96(3):315–20. 6614111 10.1016/s0002-9394(14)77821-3

[72] Howard MA, Dryja TP, Walton DS, Albert DM. Identification and significance of multinucleate tumor cells in retinoblastoma. Archives of ophthalmology (Chicago, Ill: 1960). 1989;107(7):1025-30. 10.1001/archopht.1989.01070020087037 2751457

[73] Garner A. Tumours of the retinal pigment epithelium. Br J Ophthalmol. 1970;54(11):715. 5484738 10.1136/bjo.54.11.715 PMC1215294

[74] Eagle RC Jr. High-risk features and tumor differentiation in retinoblastoma: a retrospective histopathologic study. Archives Pathol Lab Med. 2009;133(8):1203–9. 10.5858/133.8.1203 19653710

[75] Sawaguchi S, Peng Y, Wong F, Tso MO. An immunopathologic study of retinoblastoma protein. Trans Am Ophthalmol Soc. 1990;88:51–61. discussion– 2. 2095032 PMC1298577

[76] Nork TM, Millecchia LL, de Venecia GB, Myers FL, Vogel KA. Immunocytochemical features of retinoblastoma in an adult. Archives Ophthalmol (Chicago Ill: 1960). 1996;114(11):1402–6. 10.1001/archopht.1996.01100140602013 8906032

[77] Liu Y, Zhong X, Wan S, Zhang W, Lin J, Zhang P, et al. p16(INK4a) expression in retinoblastoma: a marker of differentiation grade. Diagn Pathol. 2014;9:180. 25499675 10.1186/s13000-014-0180-1 PMC4300043

[78] Theodossiadis P, Emfietzoglou I, Grigoropoulos V, Moschos M, Theodossiadis GP. Evolution of a retinoma case in 21 years. Ophthalmic surgery, lasers & imaging: the official. J Int Soc Imaging Eye. 2005;36(2):155–7. 15792319

[79] Mataftsi A, Zografos L, Balmer A, Uffer S, Stupp R, Janzer RC, et al. Chiasmatic infiltration secondary to late malignant transformation of retinoma. Ophthalmic Genet. 2012;33(3):155–8. 21526971 10.3109/13816810.2011.575431

[80] Uysal Y, Shields CL, Shields JA, Eagle RC. Jr. Malignant transformation of retinocytoma into retinoblastoma. Retinal Cases Brief Rep. 2008;2(3):256–8. 10.1097/ICB.0b013e318154b70b 25390104

[81] Kaur A, Philips CDJIIJoOO. Oculoplasty. A case report on bilateral retinocytoma. IP Int J Ocular Oncol Oculoplasty. 2020;6(2).

[82] Lam A, Shields CL, Manquez ME, Shields JA. Progressive resorption of a presumed spontaneously regressed retinoblastoma over 20 years. Retina (Philadelphia Pa). 2005;25(2):230–1. 15689823 10.1097/00006982-200502000-00025

[83] Desjardins L, Cassoux N, Matet A. Malignant Tumors of the Eye, Conjunctiva, and Orbit: Diagnosis and Therapy. In: Boffetta P, Hainaut P, editors. Encyclopedia of Cancer (Third Edition). Oxford: Academic Press; 2019. pp. 402– 13.

[84] Nadiarnykh O, McNeill-Badalova NA, Gaillard M-C, Bosscha MI, Fabius AWM, Verbraak FD, et al. Optical coherence tomography (OCT) to image active and inactive retinoblastomas as well as retinomas. Acta Ophthalmol. 2020;98(2):158–65. 31448879 10.1111/aos.14214 PMC7078953

[85] Sabir M, Jha P, Chawla R, Shaikh N. Multimodal imaging in sporadic retinocytoma. BMJ case Rep. 2023;16(2). 10.1136/bcr-2022-252260 PMC9923335 36764738

[86] Li X, Ju Y, Huang X. Multimodal Imaging of a Retinocytoma. Ophthalmol Retina. 2024;8(1):61. 37952138 10.1016/j.oret.2023.09.024

[87] Li-Wang J, Chévez-Barrios P, Thomas JS, Schefler AC. Multifocal Retinocytoma Associated with Intronic Acceptor Splice Site variants in the RB1 gene. Cureus. 2024;16(10):e70786. 39493168 10.7759/cureus.70786 PMC11531320

[88] de Alba Campomanes AG, O’Brien JM. Chapter 48 - Genetics of hereditary retinoblastoma. In: Levin LA, Albert DM, editors. Ocular disease. Edinburgh: W.B. Saunders; 2010. pp. 369–76.

[89] Grassi P, Chawla A, Rundle P. Vitrectomy for vitreous hemorrhage from vitreous operculum over Retinocytoma. Ophthalmol Retina. 2019;3(12):1055. 31810573 10.1016/j.oret.2019.08.007

[90] Shields CL, Bianciotto CG, Jabbour P, Griffin GC, Ramasubramanian A, Rosenwasser R, et al. Intra-arterial chemotherapy for Retinoblastoma: Report 2, treatment complications. Arch Ophthalmol. 2011;129(11):1407–15. 21670326 10.1001/archophthalmol.2011.151

[91] Ancona-Lezama D, Dalvin LA, Shields CL. Modern treatment of retinoblastoma: a 2020 review. Indian J Ophthalmol. 2020;68(11):2356–65. 33120616 10.4103/ijo.IJO_721_20 PMC7774148

[92] Kiratli H, Koç I. Malignant transformation of retinocytoma treated with intra-arterial chemptherapy. Can J Ophthalmol. 2016;51(3):e105–7. 27316281 10.1016/j.jcjo.2015.12.023

[93] Metz KA, Westerwick D, Driever F, Schmid KW, Le Guin CHD, Retinoblastom, Retinozytom (Retinom), editors. Der Pathologe. 2017;38(6):507– 14. 10.1007/s00292-017-0384-8 29043448

